# True type of intra-cavotricuspid isthmus re-entrant atrial tachycardia: a case report

**DOI:** 10.1093/ehjcr/ytag491

**Published:** 2026-07-10

**Authors:** Daichi Miyake, Manabu Kashiwagi, Satoshi Hata, Yusuke Teruya, Atsushi Tanaka

**Affiliations:** Clinical Engineering Center, Wakayama Medical University, 811-1, Kimiidera, Wakayama City, Wakayama 641-8509, Japan; Department of Cardiovascular Medicine, Wakayama Medical University, 811-1, Kimiidera, Wakayama City, Wakayama 641-8509, Japan; Department of Cardiovascular Medicine, Wakayama Medical University, 811-1, Kimiidera, Wakayama City, Wakayama 641-8509, Japan; Clinical Engineering Center, Wakayama Medical University, 811-1, Kimiidera, Wakayama City, Wakayama 641-8509, Japan; Department of Cardiovascular Medicine, Wakayama Medical University, 811-1, Kimiidera, Wakayama City, Wakayama 641-8509, Japan

**Keywords:** Cavotricuspid isthmus, Atrial tachycardia, Catheter ablation, Case report

## Abstract

**Background:**

Intra-isthmus re-entry, in which the tachycardia circuit is localized within the cavotricuspid isthmus region and the coronary sinus ostium, has been reported as an atypical form of atrial tachycardia. It has been delineated by three-dimensional mapping as either macro-re-entrant type around the cavotricuspid isthmus and coronary sinus area (but with the coexistence of typical atrial flutter), or focal type from the cavotricuspid isthmus area.

**Case summary:**

We present the first reported case of an apparently new form of macro-re-entrant intra-isthmus re-entry that occurred in a patient with history of prior cardiac surgery. By correcting incorrect annotation of double potentials at the cavotricuspid isthmus lesion, the local activation time map showed a re-entrant circuit rotating around a low-voltage area within the cavotricuspid isthmus region. Preoperative computed tomography revealed a pouch on the septal side of the cavotricuspid isthmus that was anatomically adjacent to the boundary of the tachycardia circuit. Termination of tachycardia was achieved by typical cavotricuspid isthmus linear ablation.

**Discussion:**

The formation of a low-voltage area, which might have been created by prior surgery, was presumed to contribute to the maintenance of intra-isthmus re-entry as a kind of boundary. By use of an ultra-high-resolution mapping system and the entrainment pacing manoeuvre, we could clearly delineate the tachycardia circuit and determine an ablation strategy.

Learning points

- Intra-isthmus re-entry, in which the tachycardia circuit is localized within the cavotricuspid isthmus region and the coronary sinus ostium, was previously reported as an atypical form of atrial tachycardia.
- By ultra-high-resolution mapping with careful manual annotation and entrainment pacing manoeuvre, the tachycardia circuit limited within cavotricuspid isthmus was clearly delineated and appropriate ablation strategy could be determined.
- The pouches sometimes play a key role in tachycardia but can be overlooked in congenital heart disease, so obtaining anatomical information by preoperative CT scans is recommended for evaluation of the tachycardia circuit, especially in cases without precise surgical records.

## Introduction

Typical atrial flutter (AFL) is widely recognized as a macroreentrant tachycardia that circulates around the tricuspid annulus. As an atypical form, there have been reports of intra-isthmus re-entry (IIR), in which the tachycardia circuit is localized within the cavotricuspid isthmus (CTI) region and the coronary sinus (CS) ostium.^1,2^ Owing to recent advantages of ultra-resolution mapping, the activation propagation of IIR can now be well visualized in detail.^3^ However in previous reports, these IIRs coexisted with typical CTI-dependent AFL, or they were delineated as focal types of atrial tachycardia (AT) by three-dimensional mapping. Here, we report the case of a patient with an apparently new form of macro-re-entrant IIR, in which the tachycardia circuit was limited to within the CTI alone.

## Summary figure

**Figure:**
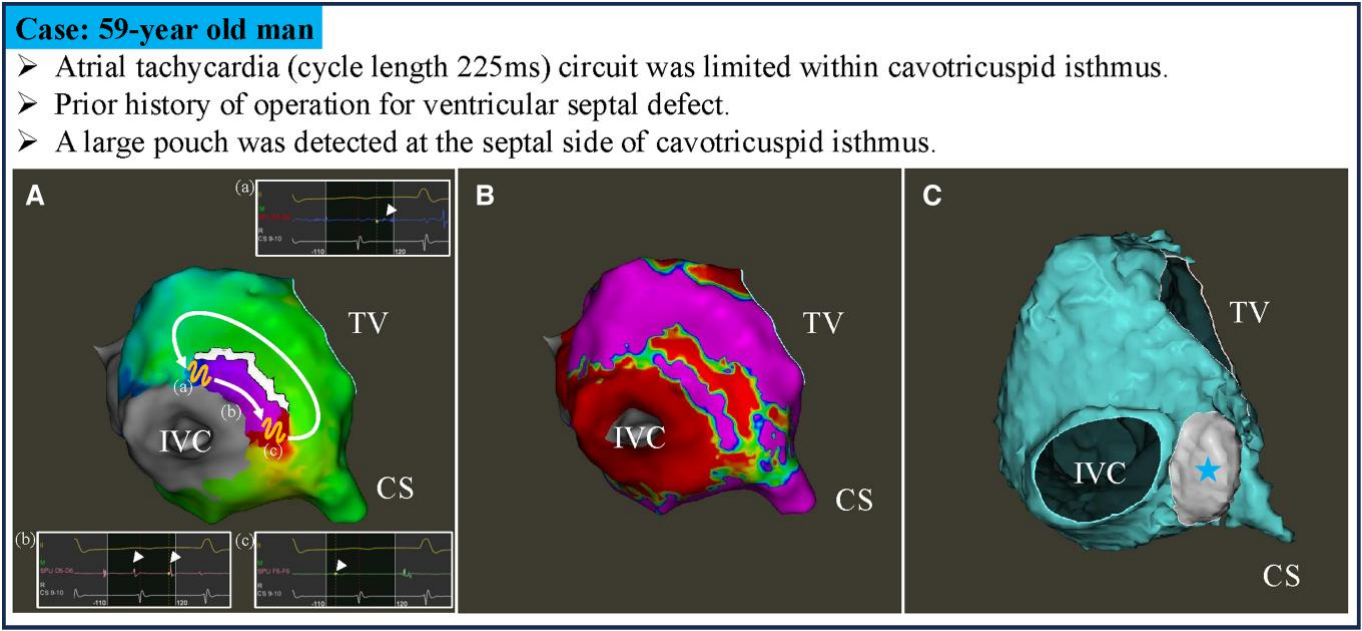
(*A*) A circuit of re-entrant atrial tachycardia was within the cavotricuspid isthmus region. Panels ‘a’, ‘b’, and ‘c’ correspond to electrocardiograms at local sites. Double potential was observed (b) and fractionated potentials were observed (a, c). (*B*) The voltage map demonstrated a low voltage area within the cavotricuspid isthmus area. (*C*) A pouch with a depth of 11.1 mm (blue star) was identified on the septal side of the cavotricuspid isthmus.

## Case presentation

**Figure 1:**
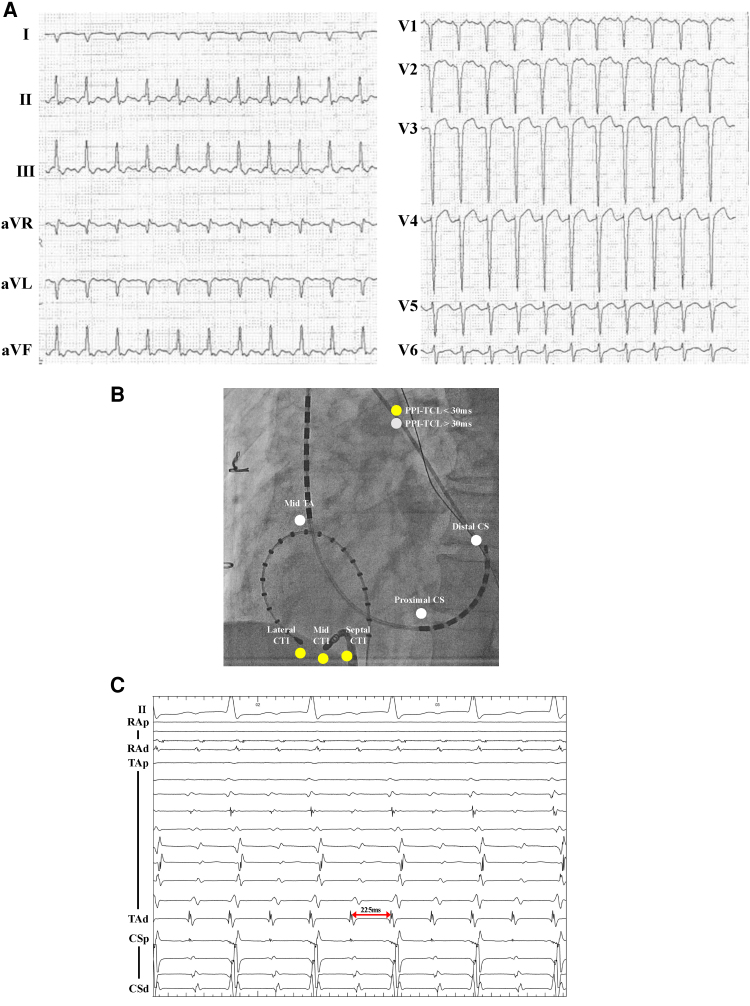
Electrocardiograms and fluoroscopy image. (*A*) The 12-lead electrocardiogram during tachycardia demonstrated sawtooth waves in leads II, III, and aVF and positive P waves in lead V1 with 2:1 atrioventricular conduction, which was similar but somewhat different from typical atrial flutter. (*B*) The fluoroscopy image and post pacing interval–tachycardia cycle length at each entrainment pacing site from left anterior oblique view. (*C*) Atrial tachycardia with a cycle length of 225 ms was induced by burst pacing, and the coronary sinus activation sequence was proximal to distal in intracardiac electrogram. CS, coronary sinus; CTI, cavotricuspid isthmus; TA, tricuspid annulus; PPI, post pacing interval; TCL, tachycardia cycle length.

Catheter ablation was planned for a 59-year-old man at our institution because of AFL with dyspnoea. He had undergone surgical repair of ventricular septal defect (VSD) in childhood, but detailed information from that time was unavailable to us. Preoperative computed tomography showed a large pouch on the septal side of the CTI and calcification in the right ventricle near the aortic annulus, which indicated a peri-membranous VSD. As shown in *Figure 1A*, 12-lead electrocardiogram showed sawtooth waves in leads II, III, and aVF and positive P waves in lead V1 with 2:1 atrioventricular conduction. This was similar to typical AFL but somewhat different in the shape of the sawtooth waves.

**Figure 2:**
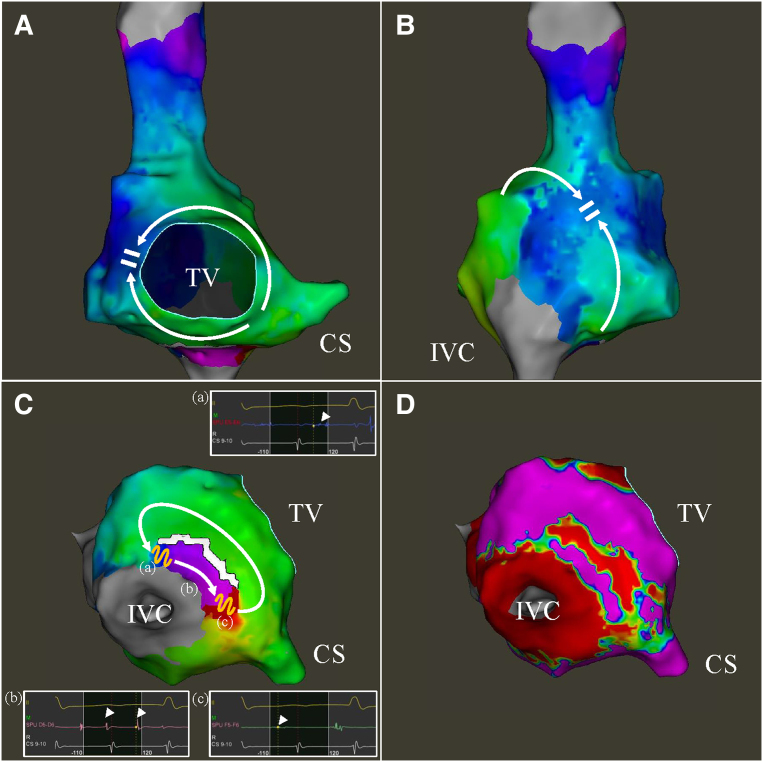
The circuit of tachycardia delineated by mapping system. (*A*) Left anterior oblique view. Typical atrial flutter circulating around the tricuspid annulus was excluded. (*B*) Posterior view. The activation wavefront demonstrated the collision of activation at the posterior right atrium. (*C*) Inferior view. A circuit of re-entrant atrial tachycardia was within the cavotricuspid isthmus region. Panels ‘a’, ‘b’ and ‘c’ correspond to electrocardiograms at local sites. The double potential was observed (b) and the fractionated potentials (66 and 63 ms) were observed (a, c). (*D*) Inferior view. The voltage map demonstrated the low voltage area within the cavotricuspid isthmus area. TV, tricuspid valve; CS, coronary sinus; IVC, inferior vena cava.

**Figure 3:**
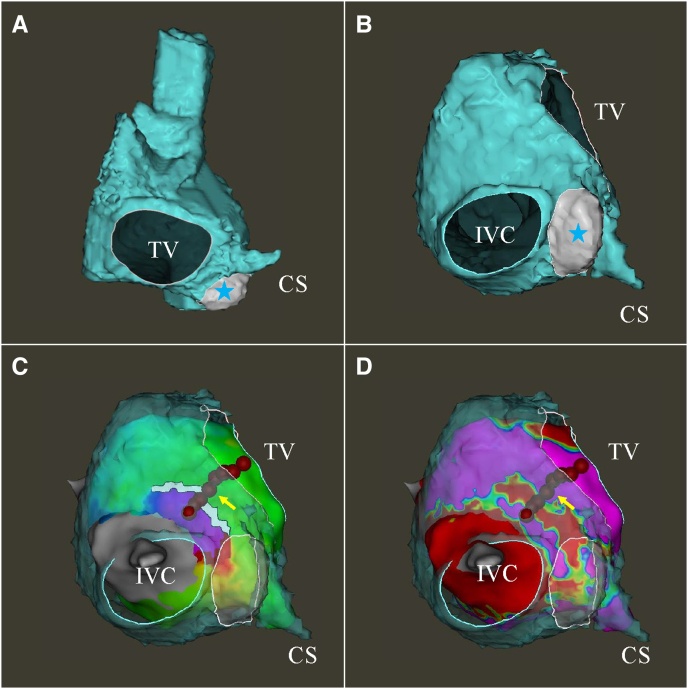
Three-dimensional construction of computed tomography. (*A*) Left anterior oblique view. A pouch with a depth of 11.1 mm (blue star) was identified in the septal side of the cavotricuspid isthmus. (*B*) Inferior view. (*C*, *D*) The tachycardia circuit and the low voltage area were anatomically adjacent to the edge of the pouch detected by computed tomography. The termination of tachycardia was achieved at the boundary site (yellow arrow). Abbreviations as in *Figure 2*.

Images from the 12-lead electrocardiogram during catheter ablation are shown in Supplementary File 1. A 20-electrode atrial cardioversion catheter (BeeAT, Japan LifeLine, Tokyo, Japan) was placed in the CS and another 20-electrode catheter (Pulscouter, Kaneka Medical Products, Osaka, Japan) was placed around the tricuspid annulus (*Figure 1B*). Intracardiac electrogram showed that the tachycardia cycle length was 225 ms (*Figure 1C*). Although the CS activation proceeded in a proximal-to-distal direction, the activation sequence of the tricuspid annulus indicated collision of activation propagation at the lateral wall of the right atrium. The post pacing interval minus tachycardia cycle length of the entrainment pacing indicated that the CTI area was involved in the tachycardia circuit, but not the anterior tricuspid annulus or the CS (Supplementary File 2). The local activation time mapping obtained by a mapping catheter (Octaray^®^, Biosense Webster, Irvine, CA) demonstrated a re-entrant circuit rotating around a low-voltage area within the CTI region (*Figure 2*). Collisions of activation wavefront at both lateral and posterior right atrium were also confirmed. Double potentials were observed at the mid CTI, and the fractionated potentials were observed at both lateral (66 ms) and septal (63 ms) sides of the CTI. The initial coherent mapping with auto-annotation mimicked focal-type AT because of incorrect annotation of double potentials at the mid CTI area (Video 1). After correction by manual annotation based on evaluation of pacing manoeuvre result, local potentials, and ripple map, coherent mapping delineated intra-cavotricuspid AT (Videos 2–5). In addition, preoperative computed tomography revealed a pouch on the septal side of the CTI that was anatomically adjacent to the boundary of the tachycardia circuit (*Figure 3*). The CTI linear ablation from the tricuspid annulus side to the boundary achieved the termination of AFL, and subsequent ablation was made from the boundary to the inferior vena cava to prevent typical AFL. The postoperative course has been uneventful for 9 months at the time of writing.

## Discussion

In previous cases of patients with IIR, fractionated or double potentials occupying approximately half of the tachycardia cycle length were observed within the CTI and/or around the ostial CS, and these characteristics were consistent with our patient’s case.^1^ IIR has previously been delineated by three-dimensional mapping as either macro-re-entrant type around the CTI and CS area (but with the coexistence of typical AFL), or focal type from the CTI area.^1–3^ In our patient’s case, entrainment pacing suggested that the anterior tricuspid annulus was outside the tachycardia circuit. Furthermore, three-dimensional mapping revealed collision of activation propagation at the lateral and posterior RA, indicating that the re-entrant tachycardia circuit existed only within the CTI. To the best of our knowledge, these characteristics are reported here for the first time.

The detailed mechanism of IIR is still unknown. Histologically, the CTI consists of a lattice structure of pectinate muscle and collagen fibre, and it has been reported that an irregular arrangement of muscle fibres may form a microreentry circuit.^4,5^ Fragmented potentials and double potentials have been recorded around the CS in cases with IIR, so a previous report hypothesized that the CS might have a central role.^1^ In the current case, the tachycardia circuit was anatomically distant from the CS, and an entrainment pacing manoeuvre indicated that the CS area was outside of the tachycardia circuit. The mechanism might therefore be different from that of previous IIRs. Interestingly, the boundary of the tachycardia circuit was continuous from the pouch at the septal CTI. Detailed records of VSD closure surgery in the patient’s childhood were unfortunately unavailable to us, but based on the historical context and the patient's testimony, cardiac surgery in hypothermia without cardiopulmonary bypass seemed to have been attempted. Although the location of incision was different from that of usual operations for peri-membranous VSD, we speculate that the boundary of tachycardia might somehow have been created by the surgical incision. However, there is still the possibility that the pouch might simply be congenital. At least, the formation of a low-voltage area is presumed to contribute to the maintenance of IIR as a kind of boundary. In patients with atrial tachycardia and congenital heart disease, anatomical pouches that were part of the arrhythmia circuit were reportedly missed in the initial mapping evaluation and subsequently corrected by careful re-mapping for pouches according to preoperative evaluation by computed tomography.^6^ In cases without adequate surgical records like this one, we suggest that obtaining anatomical information by preoperative computed tomography is useful for evaluation of the tachycardia circuit and estimation of aetiology.

Initial automated annotation misdiagnosed the tachycardia circuit because of incorrect annotation of large far-field potential at the CTI lesion. The usefulness of evaluating AT by ripple mapping rather than local activation time mapping has been reported.^7^ We therefore suggest that it is helpful to evaluate the entrainment pacing manoeuvre and annotation of local potentials in delineation of the tachycardia circuit.

## Conclusion

We reported a rare case of intra-CTI macro-re-entrant atrial tachycardia circulating around a low-voltage area within the CTI without typical AFL. By preoperative computed tomography, ultra-high-resolution mapping with careful manual annotation of local potentials and entrainment pacing manoeuvre, we could clearly delineate the tachycardia circuit and determine an ablation strategy.

## Supplementary Material

ytag491_Supplementary_Data

## Data Availability

The data that support the findings of this study are available from the corresponding author upon reasonable request.

## Lead author biography

Daichi Miyake became a clinical engineer and medical staff in Wakayama Medical University Hospital, Wakayama, Japan. He specialized in catheter ablation and cardiac implantable electronic device, especially manipulating three-dimensional mapping systems.

## Notes

**Consent:** The authors confirm that written consent for submission and publication of this case including images and associated text has been obtained from the patient in line with COPE guidance. The patient was treated in accordance with the World Medical Association Declaration of Helsinki.
