# Supplementary material for: True type of intra-cavotricuspid isthmus re-entrant atrial tachycardia: a case report
Source: Eur Heart J Case Rep. 2026 Jul 10;10(7):ytag491. doi: 10.1093/ehjcr/ytag491 (PMC13457940; doi:10.1093/ehjcr/ytag491)

**Supplementary Figure 1.**

The 12-lead electrocardiogram during catheter ablation, which was same with previous one, although lead V6 was missing.

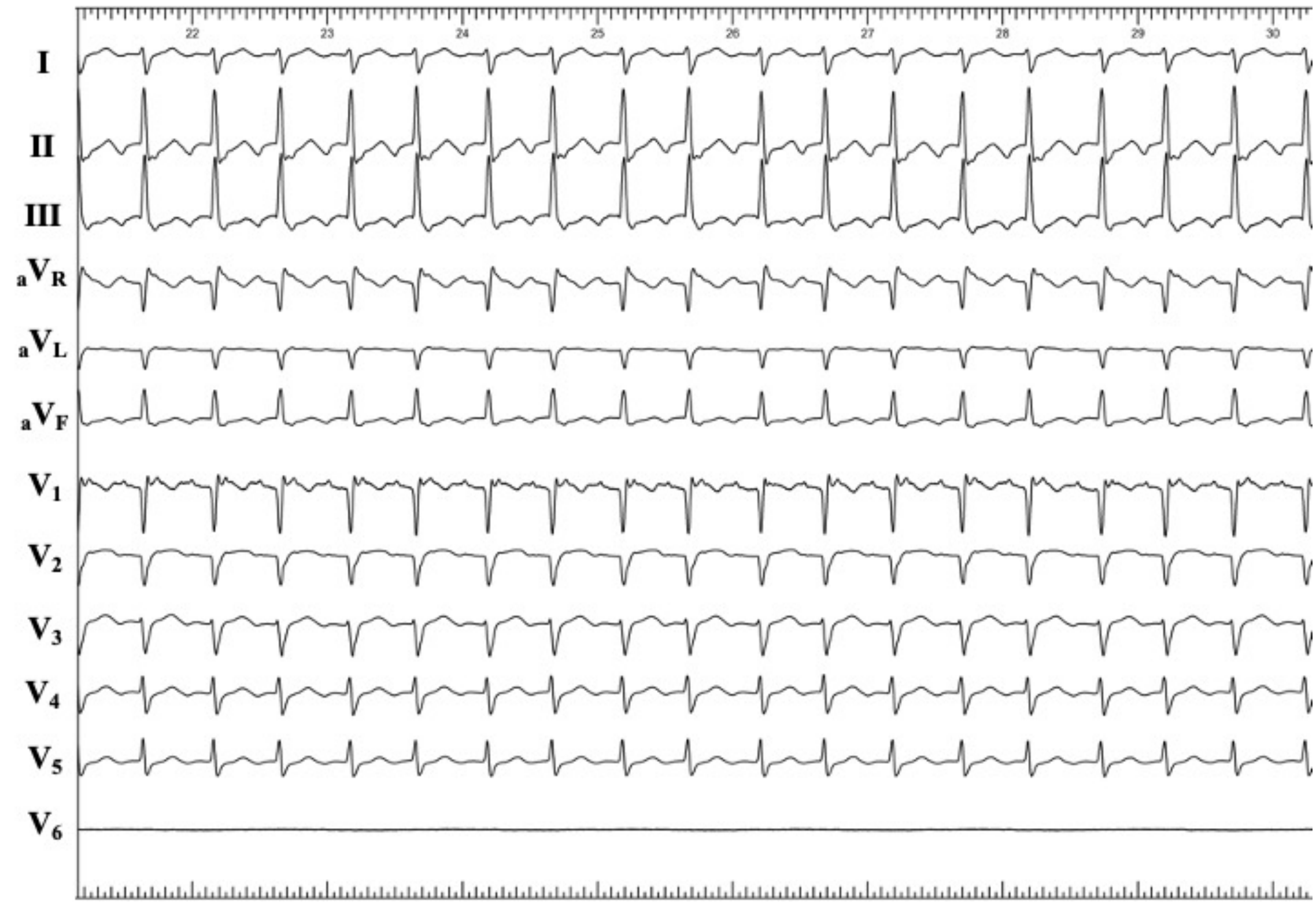

**Supplementary Figure 2A**

Pacing from lateral CTI.

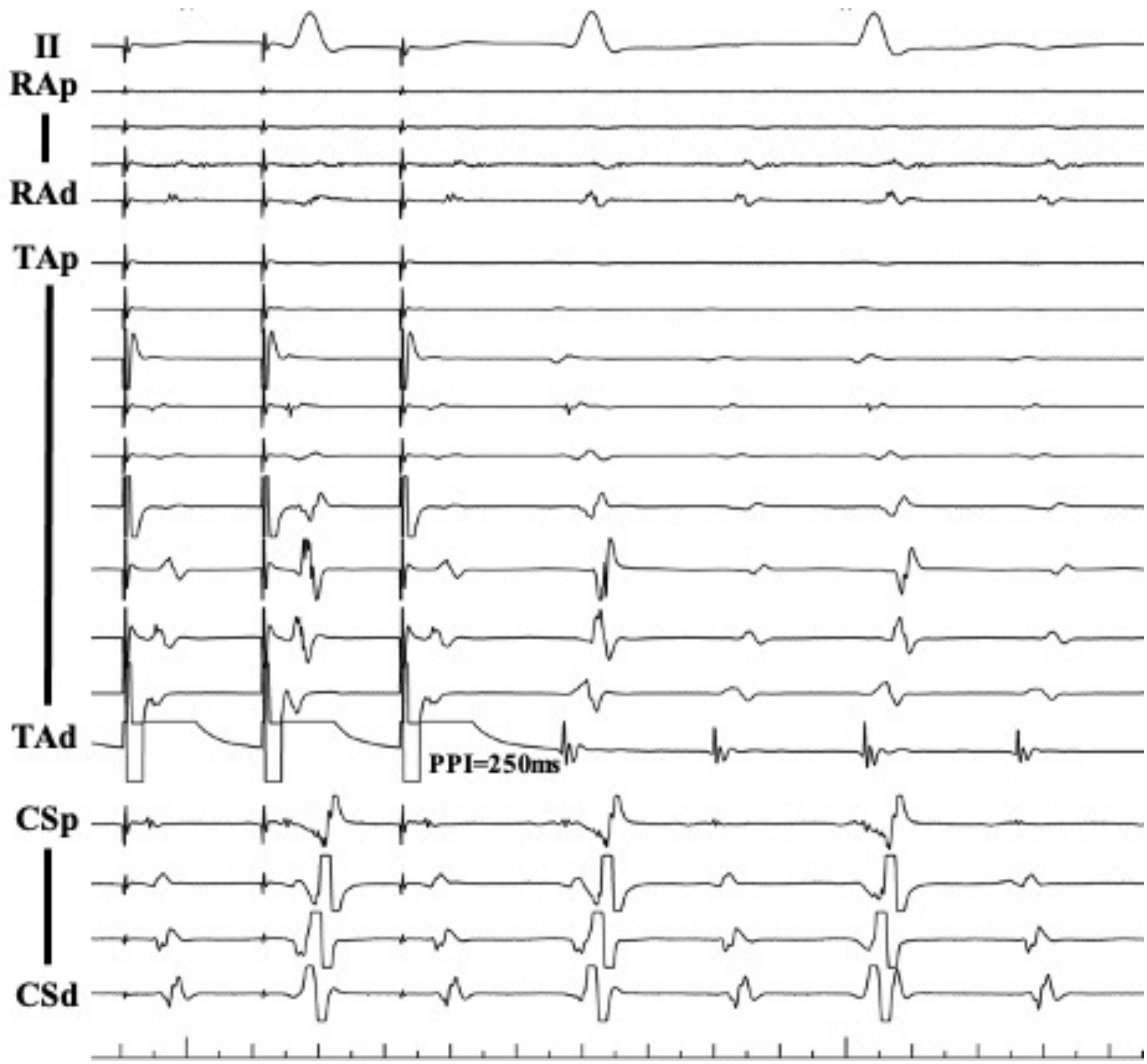

**Supplementary Figure 2B**

Pacing from mid CTI.

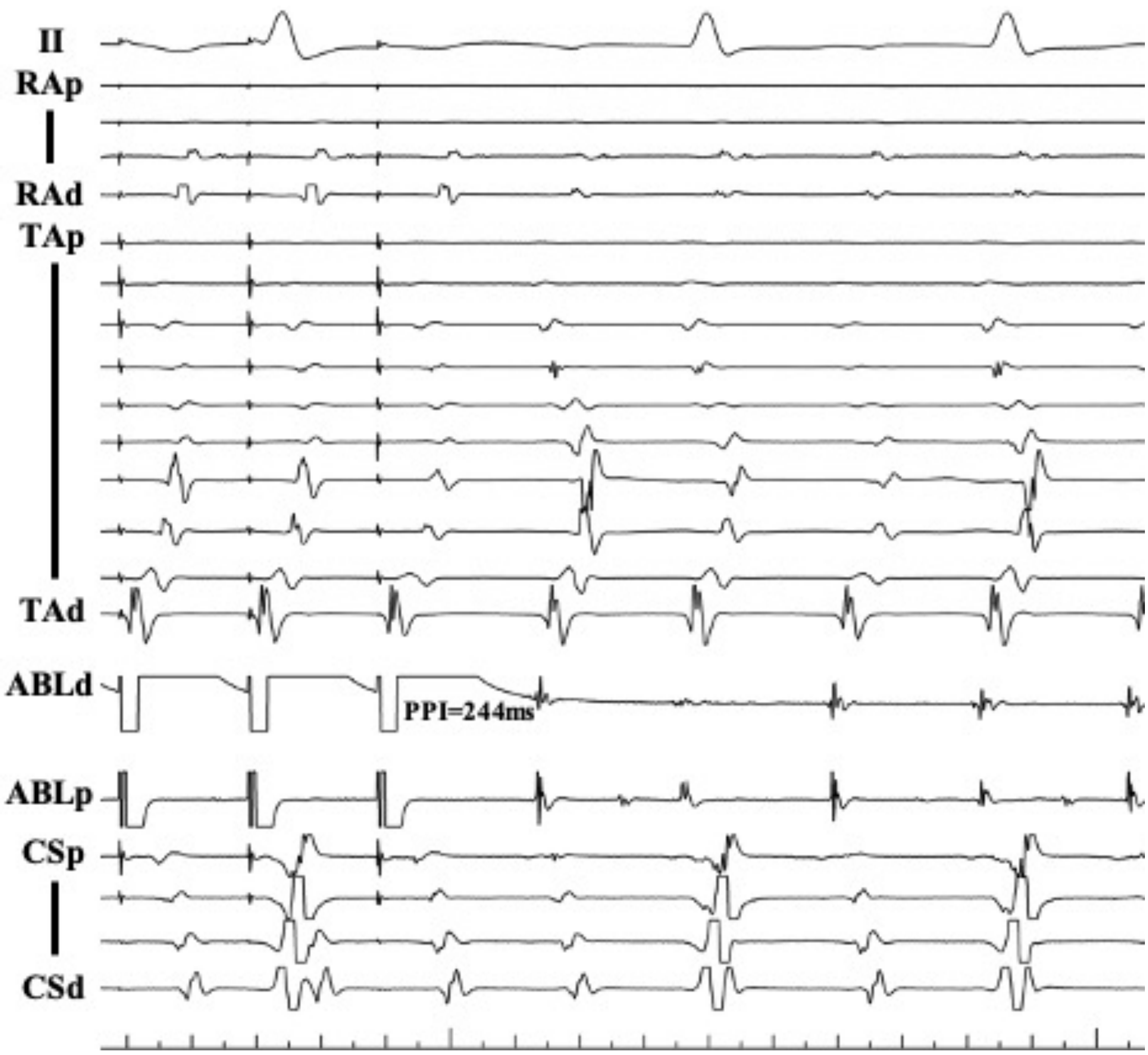

**Supplementary Figure 2C**

Pacing from septal CTI.

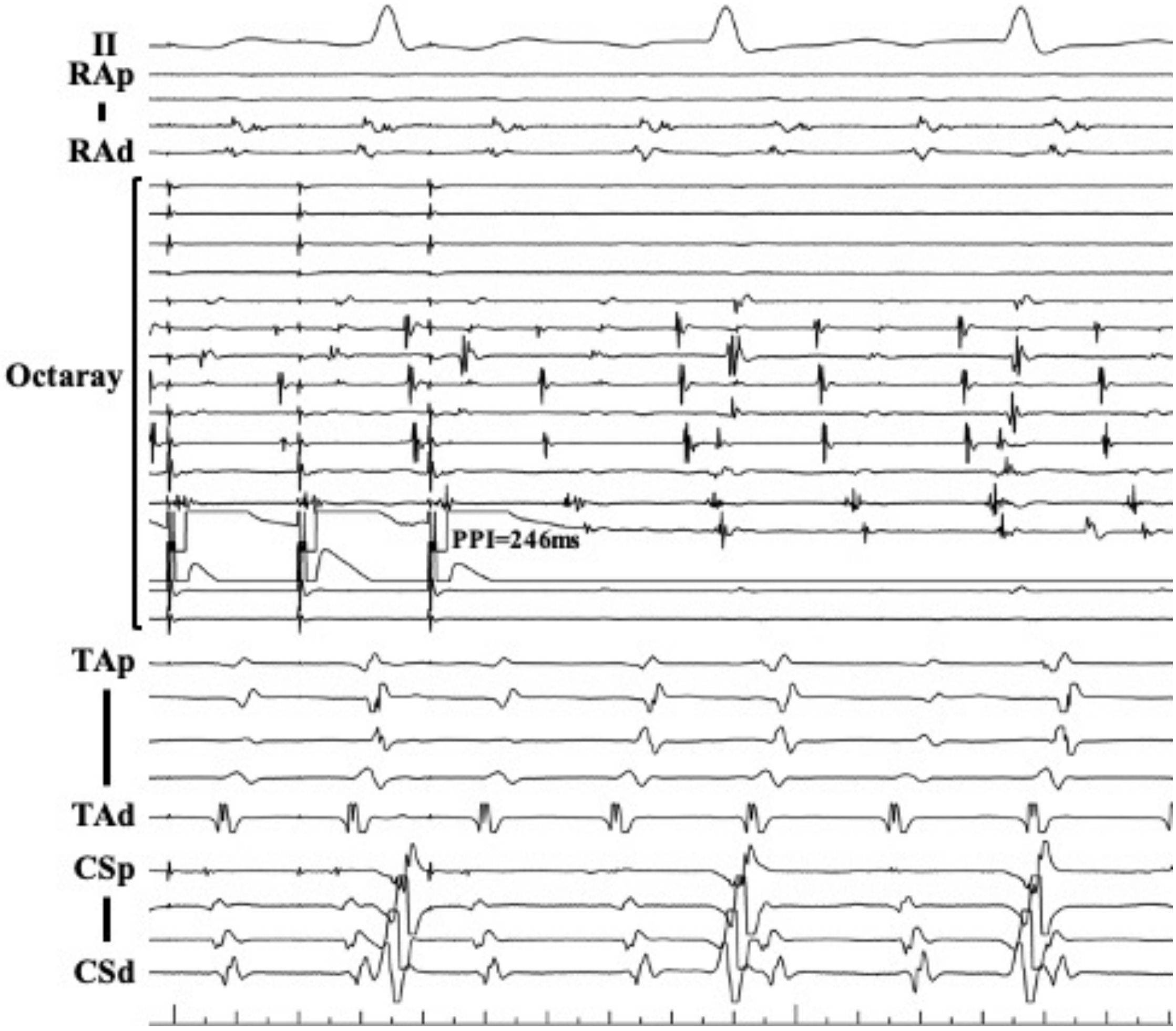

**Supplementary Figure 2D**

Pacing from proximal CS.

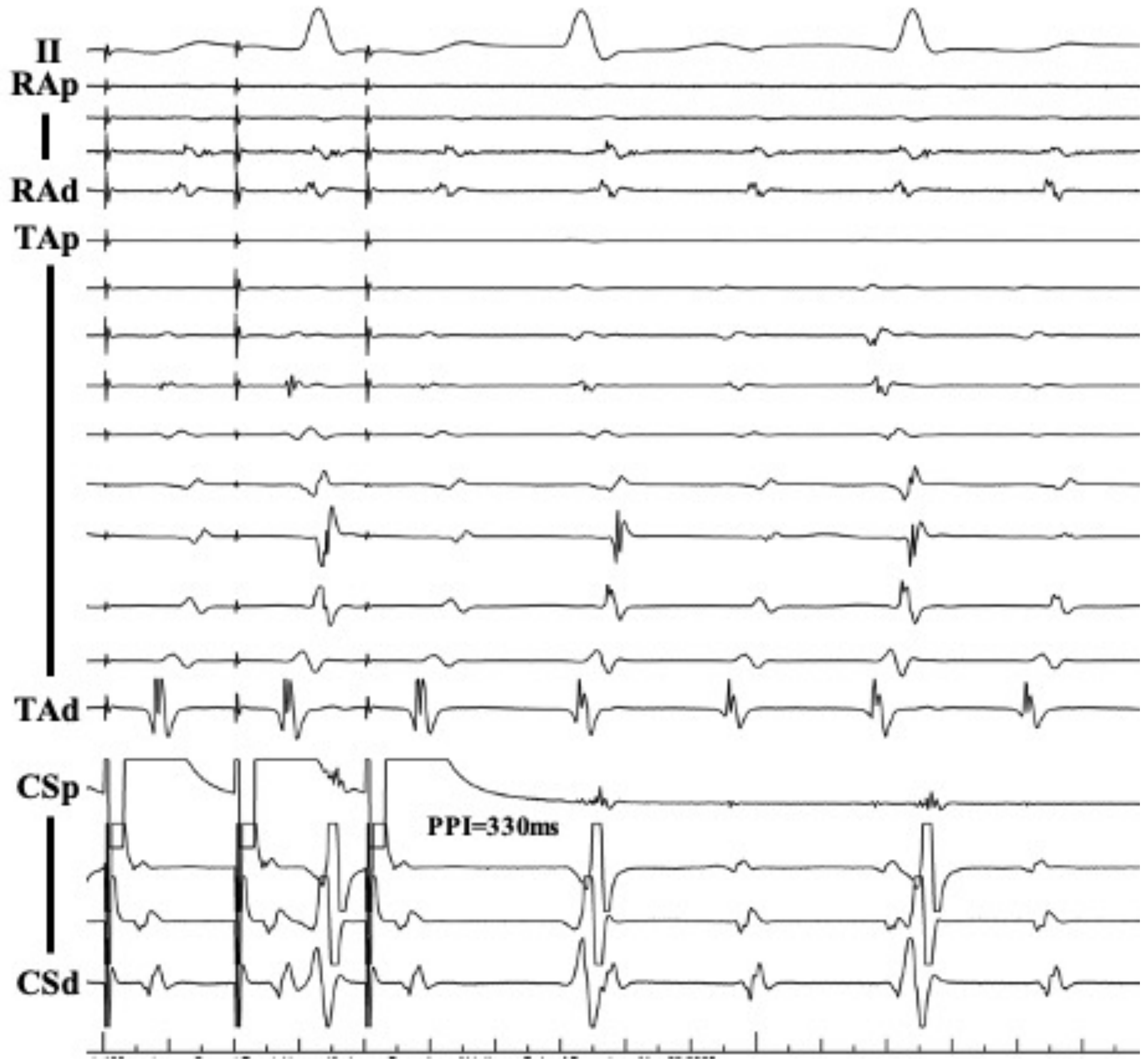

**Supplementary Figure 2E**

Pacing from distal CS.

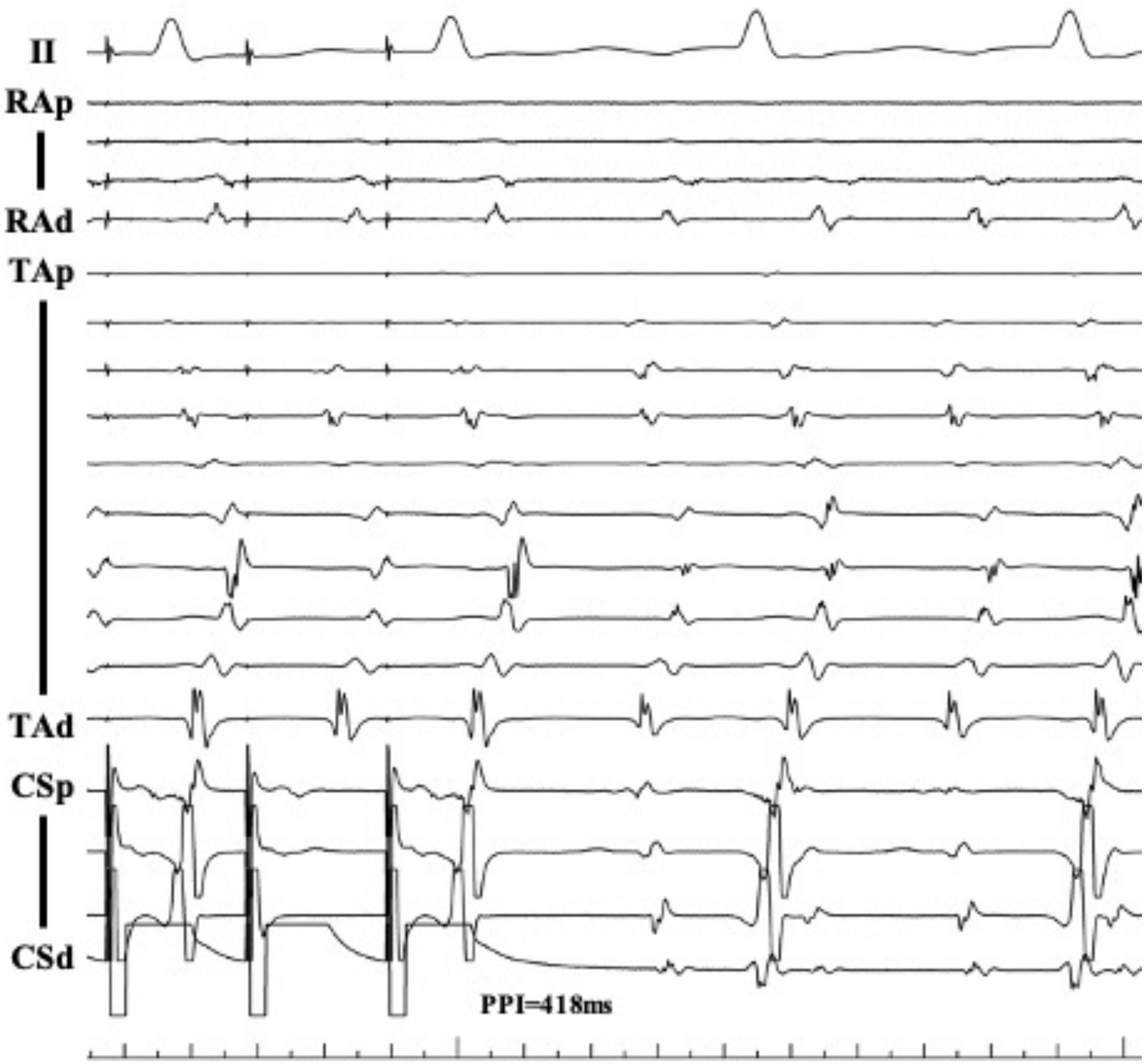

**Supplementary Figure 2F**

Pacing from mid TA.

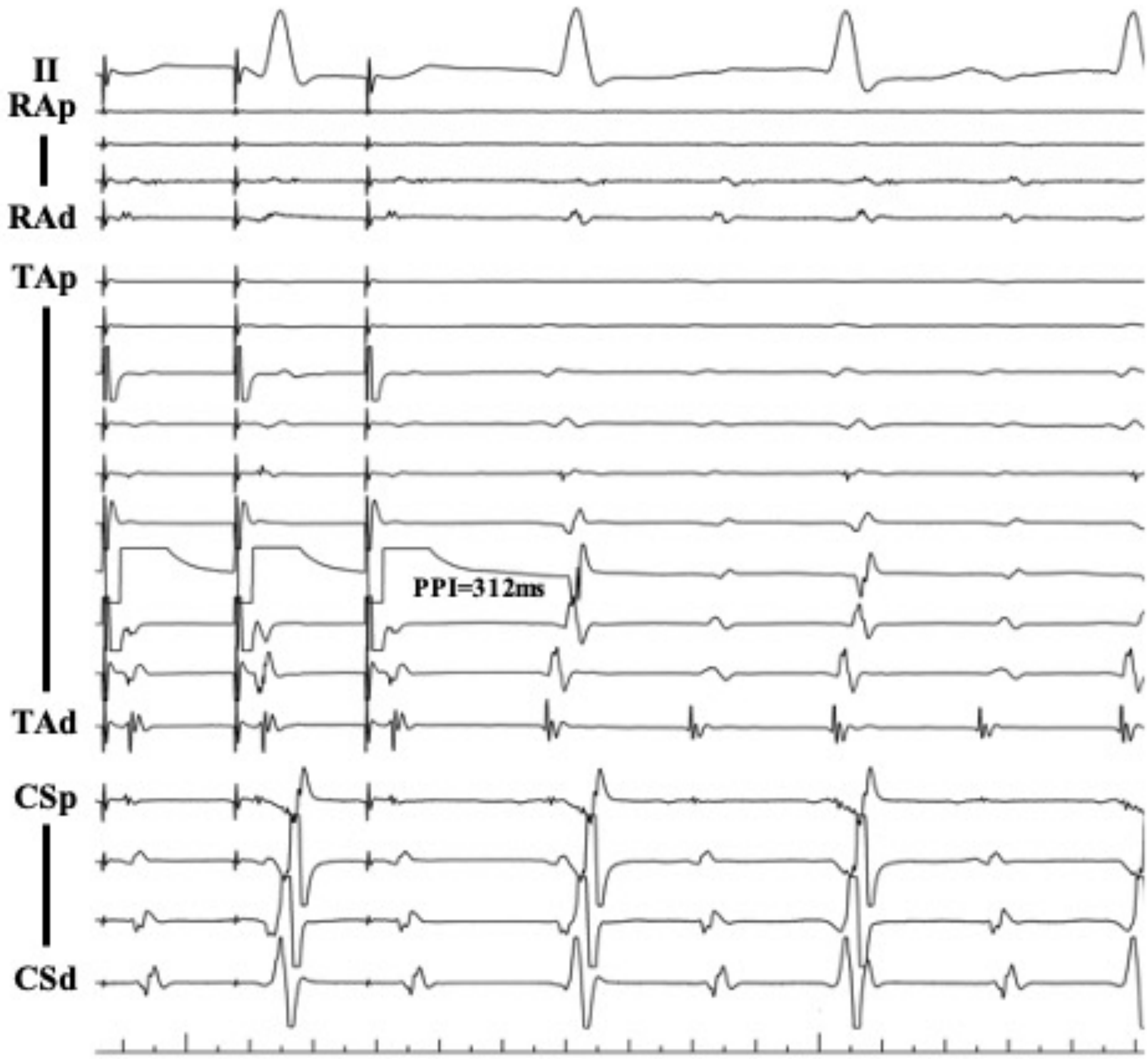

Supplement: Supplementary file 1 [file ytag491_Supplementary_Data.pdf]
